# Identification and characterization of two *Bacillus anthracis* bacteriophages

**DOI:** 10.1007/s00705-024-06005-7

**Published:** 2024-06-05

**Authors:** Lun Li, Huijuan Zhang, Haixiao Jin, Jin Guo, Pan Liu, Jiao Yang, Zijian Wang, Enmin Zhang, Binbin Yu, Liyuan Shi, Jinrong He, Peng Wang, Jianchun Wei, Youhong Zhong, Wei Li

**Affiliations:** 1 Yunnan Institute for Endemic Disease Control and Prevention, Dali, China; 2 Yunnan Key Laboratory for Zoonosis Control and Prevention, Dali, China; 3 School of Public Health, Dali University, Dali, China; 4 National Institute for Communicable Disease Control and Prevention (ICDC), China CDC, Beijing, China; 5 National Key Laboratory of Intelligent Tracking and Forecasting for Infectious Diseases, Beijing, China

## Abstract

Anthrax is an acute infectious zoonotic disease caused by *Bacillus anthracis*, a bacterium that is considered a potential biological warfare agent. *Bacillus* bacteriophages shape the composition and evolution of bacterial communities in nature and therefore have important roles in the ecosystem community. *B. anthracis* phages are not only used in etiological diagnostics but also have promising prospects in clinical therapeutics or for disinfection in anthrax outbreaks. In this study, two temperate *B. anthracis* phages, vB_BanS_A16R1 (A16R1) and vB_BanS_A16R4 (A16R4), were isolated and showed siphovirus-like morphological characteristics. Genome sequencing showed that the genomes of phages A16R1 and A16R4 are 36,569 bp and 40,059 bp in length, respectively. A16R1 belongs to the genus *Wbetavirus*, while A16R4 belongs to the genus *Hubeivirus* and is the first phage of that genus found to lyse *B. anthracis*. Because these two phages can comparatively specifically lyse *B. anthracis*, they could be used as alternative diagnostic tools for identification of *B. anthracis* infections.

**Supplementary Information:**

The online version contains supplementary material available at 10.1007/s00705-024-06005-7.

## Introduction

Anthrax is an acute zoonotic infectious disease caused by the aerobic Gram-positive and endospore-forming bacterium *Bacillus anthracis*. The spores of *B. anthracis* are resistant to extreme environmental conditions, allowing them to persist for long periods in soil [1]. Through inhalation, spores of *B. anthracis* are transported to alveolar macrophages in lymph nodes surrounding the lungs, where they germinate, and subsequent vegetative expansion causes an overwhelming flood of bacteria and toxins into the blood, killing up to 99% of untreated victims [2]. Anthrax is still considered an endemic disease in China, and a few human cases are reported annually, especially in the northern and western provinces [3].

*Bacillus cereus sensu lato* (*s. l.*) is an ecologically diverse bacterial group that comprises a growing number of species, including *B. anthracis*, *B. cereus sensu stricto* (capable of causing food poisoning and other ailments), and *B. thuringiensis* (a potential cause of severe human infections and primarily an insect pathogen that is used to control insect pests), as well as species involved in food spoilage and species used as probiotic in animal nutrition [4, 5].

Antibiotics such as penicillin G, amoxicillin, ciprofloxacin, and doxycycline are widely used in the treatment of *B. anthracis* infections [6]. However, there is a corresponding risk of low effectiveness of antibiotics against *B. anthracis* strains due to the development of antimicrobial resistance [7–9].

*B. anthracis* bacteriophages (phages) show promise for the ecological prevention and control, diagnostics, and therapeutics of anthrax. Some *B. anthracis* phages have been characterized, such as Wbeta [10], Gamma [11], Cherry [12], Fah [13], and AP631 [14]. Gamma phage is used for diagnostic purposes in the *B. anthracis* identification protocol of the Centers for Disease Control and Prevention (CDC) [15]. The Gamma phage lysis assay has been shown to be 97% specific for *B. anthracis*, but a small number of strains of other *Bacillus cereus* group members are also susceptible [16]. In China, phage AP631 was isolated from laboratory sewage in 1963 and has been used as a diagnostic phage in etiological diagnosis of anthrax [14]. Likewise, it has also been shown to lyse a small number of *B. cereus* strains [17].

In this study, *B. anthracis* phages vB_BanS_A16R1 (A16R1) and vB_BanS_A16R4 (A16R4) were isolated, and their phenotypic characteristics were identified through morphological observations and host range tests. In addition, the genomes of these two phages were analyzed and compared to closely related phages with genome sequences in the GenBank database.

## Materials and methods

### Bacterial strains and culture conditions

All bacterial strains in this study were cultured in Luria–Bertani (LB) solid medium (1.5% agar content) or LB broth medium, with shaking at 220 rpm at 37°C. The *B. anthracis* vaccine strain A16R was used as the phage indicator host strain.

### Isolation and identification of bacteriophages

Two phages (vB_BanS_A16R1 and vB_BanS_A16R4) were isolated from wet soil samples (Mountain Tuan in ICDC, Beijing, China). The isolation procedure was as follows: a soil sample (1 g) was mixed with SM buffer (10 mL) and centrifuged for 10 min at 5000 × *g*, and the supernatant was passed through a 0.22-µm filter (Millipore Beijing). Next, 500 μL of the filtrate was incubated overnight with 100 μL of host bacteria (*B. anthracis* vaccine strain A16R) at 37°C with shaking (220 rpm). Then, the supernatant was passed through a 0.22-μm filter, 200 μL of the sample was immediately mixed with 100 μL of A16R bacterial culture and vortexed vigorously, and 6 mL of molten LB soft agar (0.4%) was added and poured onto LB agar (1.5%) plates and incubated for 12 h. A single clear plaque was picked and deposited into 6 mL of LB broth containing *B. anthracis* A16R. After 6-8 hours of incubation at 37°C with shaking (220 rpm), the contents of the tube were syringe-filtered (0.22 μm), followed by a tenfold dilution and further purification by the double-agar-layer method. This purification step was repeated at least three more times to obtain a pure clone. Finally, the purified phages were propagated on the host A16R and used in subsequent experiments. SM buffer (5.8 g of NaCl, 2.0 g of MgSO_4_, 50 mL of 1 M Tris, pH 7.5, and 5 mL of 2% gelatin per liter) was used for phage concentration and storage.

### Transmission electron microscopy

Referring to previous methods for transmission electron microscope observation [18], the purified phages were dropped onto 400-mesh carbon-coated copper grids for 15 min and negatively stained with 2% phosphotungstic acid (PTA, pH 6.5) for 30 s, and the morphology of the phages was observed by transmission electron microscopy (Hitachi HT7700, Japan, 80 kV) with a Gatan 832.10 W CCD camera (Gatan, USA). Using Gatan Digital Micrograph software, the size of the phage particles was estimated based on the average of three measurements.

### Determination of the host spectrum

A total of 90 strains of *Bacillus* bacteria (Supplementary Table S3), including five virulent *B. anthracis* strains, were used in the host range analysis. These strains included the *Bacillus cereus s.l.* members *B. cereus*, *B. thuringiensis*, *B. mycoides*, *B. pseudomycoides*, *B. cytotoxicus*, *B. weihenstephanensis*, and *B. wiedmannii*. Ten-microliter aliquots of undiluted phage (titer >10^8^ PFU/mL) were dropped onto double-layer agar premixed with 200 μL of bacterial culture and incubated at 37°C for 12 h. The phage sensitivity of the bacteria was assessed based on the presence of lysis plaques. Phage AP631 served as a control in this experiment.

### Determination of biological characteristics of the phage

To determine the optimal multiplicity of infection (MOI), we mixed phages and the host bacterium *B. anthracis* A16R at various ratios, and the titers were calculated using a double-layer agar plate assay after 6 h of incubation.

A one-step growth curve assay was performed as described previously [19] with slight modifications. Sampling started at time zero and occurred once every five minutes during the first 20 minutes, and then once every 10 minutes up to 110 min. The stability of phages was determined by measuring phage titers at various temperatures (4°C, 28°C, 37°C, 50°C, 60°C, and 70°C) and pH values (pH 3-13). For these stability experiments, 1-mL phage lysates (> 10^8^ PFU/mL in LB broth medium) were incubated at the respective temperatures for 60 min, while for pH stability, phage lysates (> 10^8^ PFU/mL in LB broth medium) were mixed with different pH solutions at a ratio of 1:1000 (to reduce the effect on pH) for 60 min. Finally, a plaque assay was performed on *B. anthracis* A16R at 37°C. Phage AP631 served as the control in this experiment.

### Genomic DNA extraction, sequencing, annotation, and analysis

Phage DNA extraction was performed as described previously [20], using a λ Phage Genomic DNA Extraction Kit (ABigen Corp., China), and the DNA samples were randomly fragmented using a Bioruptor Pico Non-Contact Ultrasonic Nucleic Acid Breaker to produce DNA fragments of the needed length for sequencing. Then, a library was constructed using an ALFA-SEQ DNA Library Prep Kit according to the instructions, and PE150 sequencing was performed on the library using an Illumina Nova 6000 platform. Soapnuke software (v2.0.5) [21] was used for quality control. *De novo* assembly of clean data was performed using Megahit (v1.1.2) [22] with default parameters. Finally, the remaining gaps between the scaffolds were filled using PCR and Sanger sequencing. The BLASTn (https://blast.ncbi.nlm.nih.gov/Blast.cgi, accessed on 7 July 2023) tool was used to identify related phage sequence in the GenBank database. Phylogenetic analysis was performed by the neighbor-joining method in MEGA11 [23] and Clustal W [24] based on whole-genome sequences, using iTOL [25] for visualization.

Assembled phage sequences were annotated automatically using the RAST (Rapid Annotation using Subsystem Technology) website (https://rast.nmpdr.org/, accessed on 7 July 2023) [26], based on the Subsystems database and the FIGfams database. Afterwards, the predicted genes were verified using the BLASTp (https://blast.ncbi.nlm.nih.gov/Blast.cgi, accessed on 7 July 2023) tool on the NCBI website (https://www.ncbi.nlm.nih.gov). The genomic map was drawn using SnapGene 4.2.4., and the alignment results were visualized using Easyfig2.2.5 [27]. Adobe Illustrator 2020 was used to adjust the colors and characters. Antibiotic resistance genes and virulence factors were predicted using CARD [28] (https://card.mcmaster.ca/, accessed on 7 July 2023) and VFDB [29] (http://www.mgc.ac.cn/VFs/main.htm, accessed on 7 July 2023). Finally, the Conserved Domain Search Service [30] in NCBI and CD Search online (http://wwwncbinlm.nih.gov/Structure/cdd/wrpsb.cgi, accessed on 7 August 2023) were used to identify conserved domains within the encoded viral proteins.

### Accession numbers

The whole-genome sequences were deposited in the GenBank database under the accession numbers OR509663 (vB_BanS_A16R1, A16R1) and OR509662 (vB_BanS_A16R4, A16R4).

## Results

### Morphology and host spectrum of bacteriophages

**Fig. 1:**
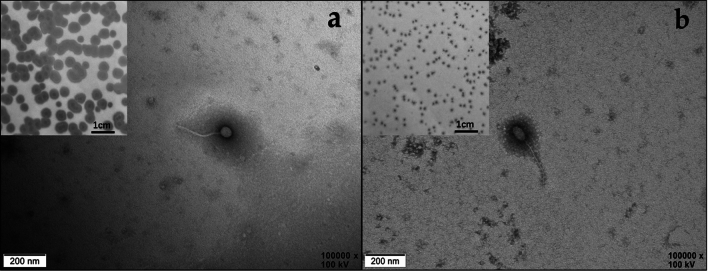
Transmission electron micrographs and plaque morphology of A16R1 (**a**) and A16R4 (**b**)

Plaques formed by A16R1 and A16R4 exhibited different morphologies, and the plaques and halos of A16R1 were larger than those of A16R4 (Fig. 1). A16R1 has a typical icosahedral head (53 ± 5 nm) and a long noncontractile tail (200 ± 8 nm), whereas A16R4 has an elongated head (40 ± 2 nm × 81 ± 5 nm) and a long noncontractile tail (270 ± 18 nm). Both phages belong to the class *Caudoviricetes* (Fig. 1).

### Host spectrum determination of phages A16R1 and A16R4

Both A16R1 and A16R4 formed clear plaques on five virulent *B. anthracis* strains. In addition, turbid plaques were formed on some *B. cereus* and *B. thuringiensis* strains. We also found that A16R4 could lyse a strain of *B. mycoides* (BMY02), forming clear plaques. The phages differed in their host range, but both infected *B. anthracis* strains (Table 1).

**Table 1:**
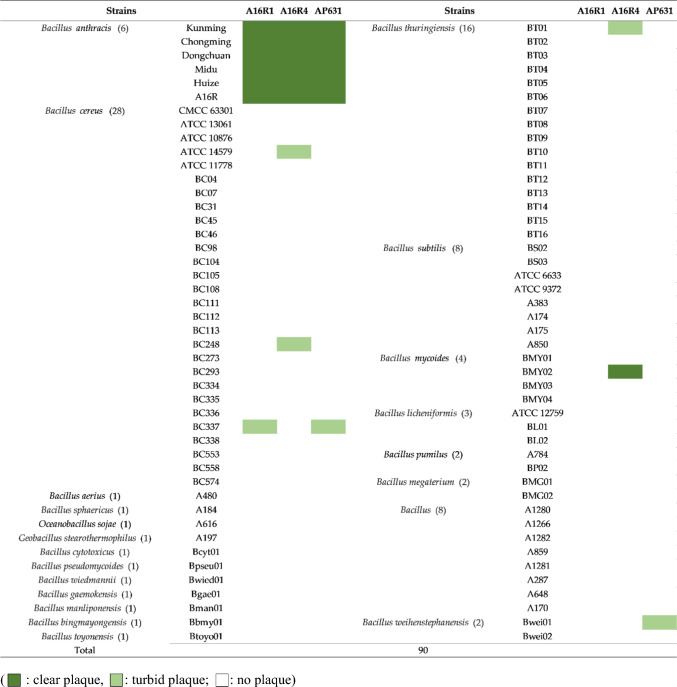
Host range of phages A16R1, A16R4, and AP631

### Optimal multiplicity of infection, one-step growth, and stability

The optimal MOI of phage A16R1 was 0.01, whereas that of phage A16R4 was 0.001. In addition, the two phages produced different one-step growth curves, including latent and burst phases (Supplementary Fig. S1). Both phages showed high stability when kept at 4°C, 28°C, 37°C, or 50°C for 60 min. Phage A16R4 was quickly inactivated at temperatures above 50°C, whereas the activity of phage A16R1 began to decrease at 50°C, and at 60°C, a very small number of phages were still active after 60 min (750 PFU/mL). In pH stability tests, both phages A16R1 and A16R4 could survive at pH 6–9; and, phage remained active for up to 60 min at pH 10 (Supplementary Fig. S1).

### Genomic characteristics of phages A16R1 and A16R4

The genomes of A16R1 and A16R4 are 36,569 bp and 40,059 bp in length, respectively. Their G+C content (35.0%) is similar to that of their bacterial host *B. anthracis* A16R vaccine strain (35.4%) [31], and no rRNA or tRNA genes were identified. The number of predicted proteins was 56 and 59, respectively. The genome sequences were screened online using the CARD and VFDB databases, and no antibiotic resistance genes or virulence factors were found.

**Fig. 2:**
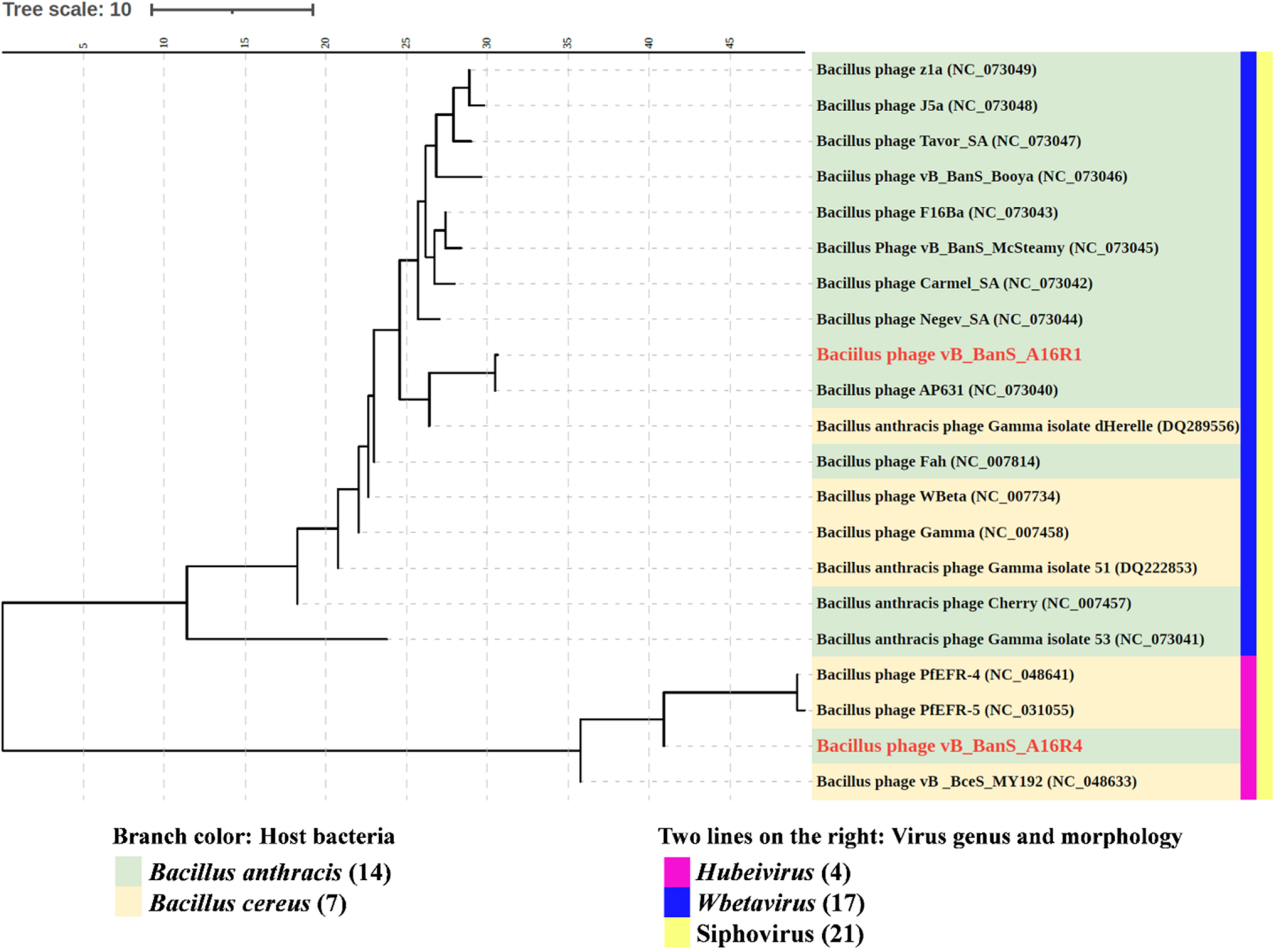
Phylogenetic analysis of phages A16R1 and A16R4 and their closest relatives based on whole-genome sequences. iTOL [25] was used for visualization of the tree.

The genomes of phages A16R1 and A16R4 differed greatly, with only 13% coverage and 83.19% sequence identity in the similar regions. Using BLASTn algorithm tools, phylogenetic relationships based on the whole-genome sequence showed that the two phages belong to different clades. Phage A16R1 is closely related to members of the genus *Wbetavirus*, while phage A16R4, together with three *B. cereus* phages (PfEFR-4, PfEFR-5, and MY192), formed a clade in the genus *Hubeivirus* (Fig. 2).

**Fig. 3:**
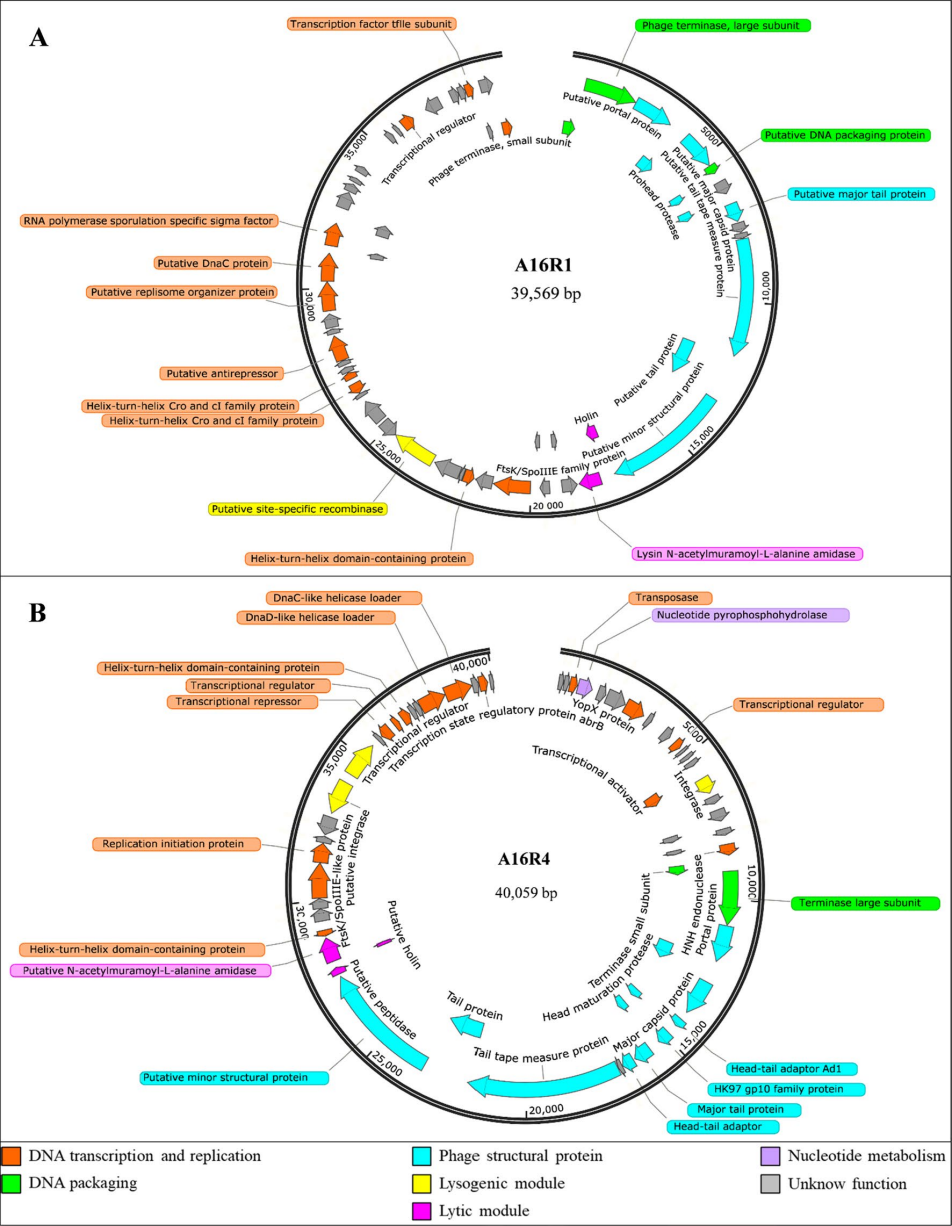
Genome maps of phages A16R1 (**A**) and A16R4 (**B**) showing their encoded proteins and predicted functions

According to predicted protein function, including DNA packaging, DNA transcription and replication, phage structure, lytic module, lysogenic module, nucleotide metabolism, and unknown functions (Fig. 3), seven different functional units were identified. In the lytic module, holin (ORF16) and lysin N-acetylmuramoyl-L-alanine amidase (ORF17) were predicted in A16R1 (Supplementary Table S1), and a putative holin (ORF38) and putative N-acetylmuramoyl-L-alanine amidase (ORF39) were predicted in A16R4 (Supplementary Table S2). A lysogenic module containing a putative site-specific recombinase (ORF27) similar to members of the serine recombinase family (cd0038) was found in A16R1. This type of integrase in the serine family is larger than others, uses a catalytic serine for strand cleavage, recognizes shorter attachment site *attP* sequences, and does not require host cofactors [32]. A16R4 was predicted to encode two integrases (ORF15 and ORF47) and a protein-encoded transcriptional regulator (ORF48) containing a domain of the AimR family lysis-lysogeny pheromone receptor (NF038010). AimR is the intracellular pheromone receptor that is responsible for the choice between lysis and lysogeny [33]. In addition, an auxiliary metabolic gene (AMG) [34] was predicted in A16R4 (ORF4).

**Fig. 4:**
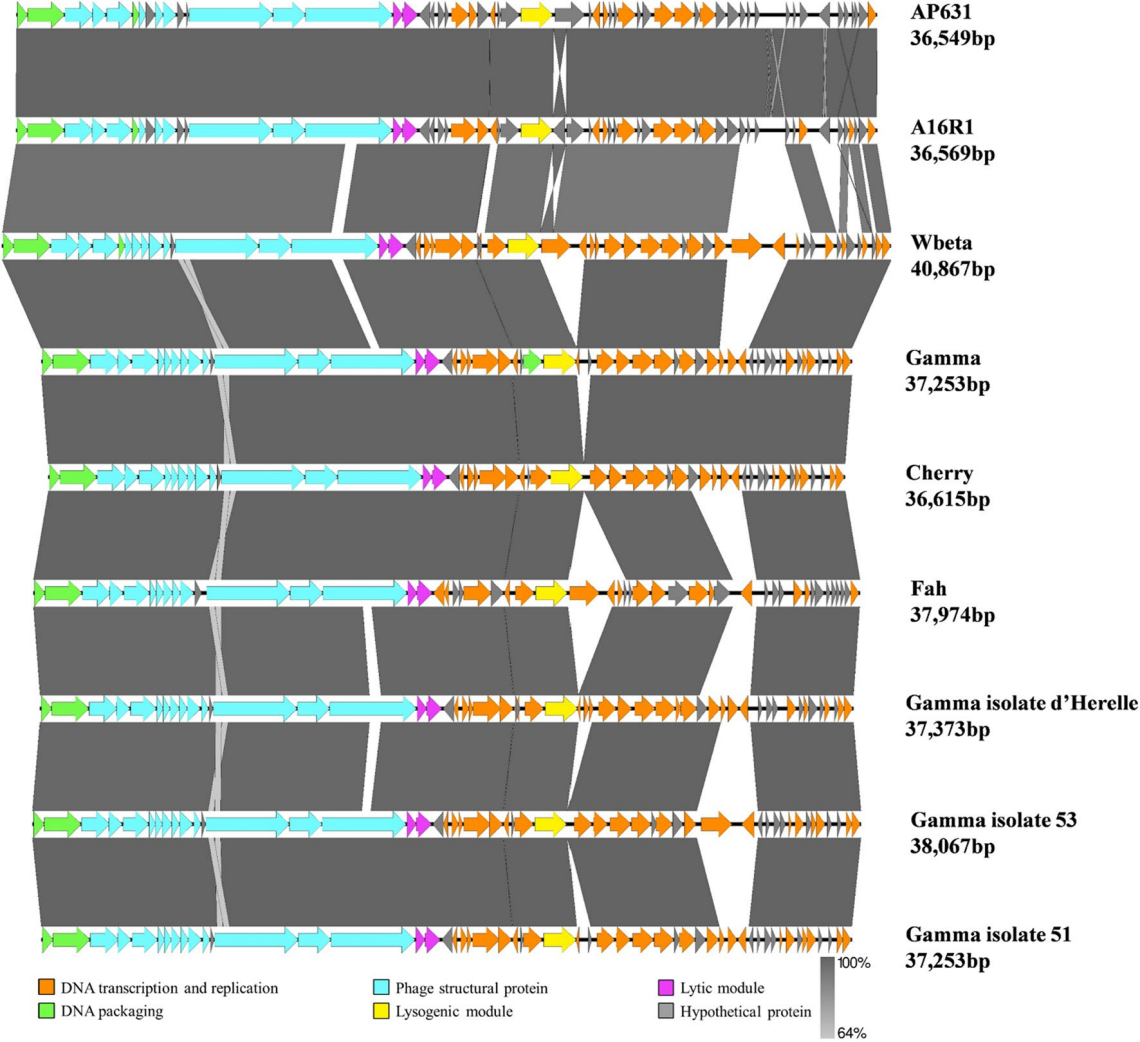
Genome collinearity of phages A16R1, AP631, Wbeta, Gamma, Cherry, Fah, Gamma isolate d'Herelle, Gamma isolate 53, and Gamma isolate 51

The upstream region of the A16R1 genome (Fig. 4), containing genes encoding DNA packaging components, structural proteins, and components performing host lysis functions, is similar to the typical arrangement found in members of the genus *Wbetavirus*. The downstream region of the genome contains genes involved in viral replication and encoding numerous predicted transcription factors that are likely to regulate viral gene expression [13]. When comparing A16R1 with AP631, we found that they had 99.98% sequence identity with 99% coverage, and the largest difference was that the ORF28 gene in A16R1 was reversed in its orientation with respect to its counterpart in AP631 (Supplementary Fig. S2).

**Fig. 5:**
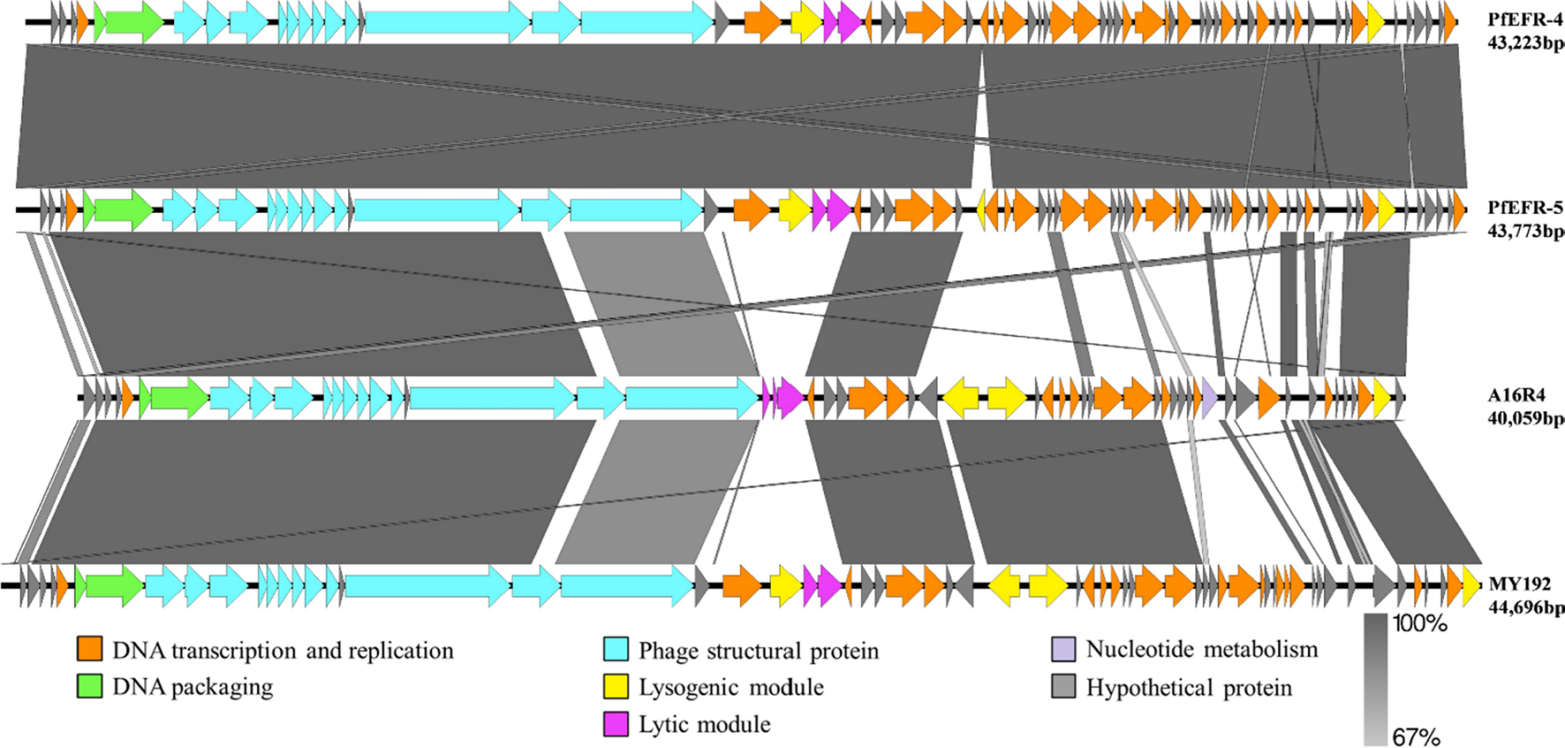
Comparative genome analysis of phages A16R4, PfEFR-4, PfEFR-5, and MY192

Compared to other members of the genus *Hubeivirus*, A16R4 exhibited only 84% coverage with MY192 and 65% coverage with PfEFR-4 and PfEFR-5, but in most of the covered regions, the sequence identity was greater than 99%. In the similar regions (indicated in Fig. 5 by shading between the genome diagrams) most of the genes were predicted to encode proteins involved in DNA packaging and structural proteins. The sequences of the integrase (ORF47) and transcriptional regulator (ORF48) of A16R4 were identical to those of MY192, and another integrase (ORF15) was identical to that of PfEFR-5. Notably, A16R4 has a lysis module that is unique in this genus, encoding a putative peptidase (ORF37) and a putative N-acetylmuramoyl-L-alanine amidase (ORF39), which are identical to those of *B. anthracis* phage vB_BanS_Athena, while the putative holin (ORF38) in A16R4 is 93.62% identical to those of *B. thuringiensis* phage BtCS33 (Supplementary Table S2).

## Discussion

In this study, we isolated and characterized two *B. anthracis* phages: A16R1 and A16R4. Electron microscopy showed that both phages belonged to the class *Caudoviricetes* and had siphovirus-like morphological characteristics (Fig. 1). With extended culture time, we observed halo phenomena on phage plaques in the bacterial lawns. Previous research showed that such halos are associated with the presence of phage-associated exopolysaccharide (EPS) depolymerases [35]. In support of this, we found that such depolymerases were predicted to be present in the tail structure of A16R1 (ORF15) and A16R4 (ORF36), and their function is inferred to be penetrating EPS layers [36, 37].

Host spectrum assays showed that A16R1 and A16R4 had relatively specific lytic ability for *B. anthracis* (5/5, 100%), but they exhibited the ability to lyse individual strains of other species (mostly *B. cereus*), resulting in turbid plaques. Generally, the phage host range is determined by specific receptor structures [38]. In siphophages, receptor-binding proteins are generally associated with viral tail structures, such as straight tail fibers or spikes attached to the tail or baseplate [38, 39]. Accordingly, after comparing the tail structures of A16R1 and A16R4 (Supplementary Fig. S3), we inferred that the host range differences between these phages might be due to heterogeneity in the viral tail structure. In addition, higher specificity of A16R1 than AP631 for *B. anthracis* was observed, suggesting that A16R1 might be used as an alternative or replacement for *B. anthracis* diagnostic phages in the future.

According to the bacteriophage classification standard [40], A16R1 should be classified as a member the genus *Wbetavirus*. Phylogenetic analysis showed that A16R1 is located in the same branch as phage AP631, with 99.98% sequence identity. The main difference between these phages is that the ORF28 of A16R1 is in the reverse orientation. This difference might have been a result of the sequencing procedure, but since the function of ORF28 has not yet been determined, the differences cannot be evaluated. This would suggest that phage A16R1 should be considered an isolate of phage AP631. The presence of a lysogenic module similar to those found in most of the proposed members of the genus *Wbetavirus*, such as Wbeta phage [19], suggests that A16R1 is a temperate phage. Nevertheless, although the phages AP631 and Gamma have lysogenic modules in their genomes, they are still used as *B. anthracis* diagnostic phages by the CDC in China and the United States, respectively, due to their rapid and highly specific lytic characteristics.

A16R4 belongs to the genus *Hubeivirus* and is, to our knowledge, the first member of the genus *Hubeivirus* found to infect *B. anthracis*. Three other phages in this genus, PfEFR-4, PfEFR-5, and MY192, were detected and isolated in their natural hosts. The lysogenic modules of A16R4 were completely identical to those of the other three phages. It is therefore possible that A16R4 was previously present as a prophage in certain bacteria and that it was subsequently excised from the bacterial genome and entered the lytic cycle [41]. The lysogenic characteristics of A16R4 need to be investigated in the future. Notably, the unique lytic module in A16R4 did not exhibit any similarity to those of the other three phages of the genus *Hubeivirus*, and its putative N-acetylmuramoyl-L-alanine amidase (ORF39) was completely identical to that of *Bacillus* phage vB_BanS_Athena (Supplementary Table S2), which encodes an endolysin, PlyB, that had been identified in myovirus phage Bcp1 [5]. In addition, phage A16R4 has a nucleotide metabolism module (ORF4). This module contains an AMG, a phage-encoded and host-derived metabolic gene that enables host cells to stop programmed cell death, which improves survival in a nutrient-depleted environment and eventually increases viral replication [34]. Recently, a study revealed that AMGs are abundant in soil viruses and might encode enzymes that are involved in degradation of complex polysaccharides [42]. Whether phage A16R4 isolated from the soil also increases its own replication or encodes the corresponding enzyme due to the presence of the AMG (ORF4) remains to be studied.

Ideally, strictly lytic phages are the main choice for phage therapy [43, 44]. The two *B. anthracis* phages identified in this study, due to the possibility of entering the lysogenic pathway, do not fully meet the requirements for use in clinical therapeutic applications. However, temperate phages can also be exploited to control bacterial infection based on their ability to impair bacterial group behaviors upon infection and/or lysogenization [43]. Furthermore, through genetic engineering in lysogenic hosts, such phages can be altered to have a wider host range [45], allowing these phages to survive better in the environment than they would if they could only use *B. anthracis* as a host [46].

## Perspectives

In this study, two *B. anthracis* phages (A16R1 and A16R4) belonging to the genera *Wbetavirus* and *Hubeivirus*, respectively, were identified and characterized. Their temperature and pH stability, their short latent and burst periods, and their specificity for *B. anthracis* indicate their potential to be used as an alternative or replacement diagnostic phage for *B. anthracis*. In the future, more systematic stability tests under different environmental conditions are necessary. In addition, due to their biological characteristics and genome features, these two phages have potential as microecological biocontrol agents in phage cocktails to eliminate *B. anthracis* in the environment or in anthrax-associated outbreaks or in bioterrorism scenarios. However, more detailed scientific research is still needed, including receptor identification and assessment of their effect on pathogenicity. In addition, finding a simple way to induce the germination of *B. anthracis* spores could increase phage efficacy, as only vegetative bacteria of *B. anthracis* can be eliminated by phages.

### Supplementary Information

Below is the link to the electronic supplementary material.

Supplementary file1 (DOCX 514 KB)

## Data Availability

The datasets generated and/or analysed in the current study are available from the corresponding author on reasonable request.
