# Supplementary material for: Identification and characterization of two Bacillus anthracis bacteriophages
Source: Arch Virol. 2024 Jun 5;169(7):134. doi: 10.1007/s00705-024-06005-7 (PMC11150296; doi:10.1007/s00705-024-06005-7)
Supplement: Supplementary file 1 — Supplementary file1 (DOCX 514 KB) [file 705_2024_6005_MOESM1_ESM.docx]

Identification and characterization of two *Bacillus anthracis* bacteriophages

Lun Li^1,2,3,4,5†^, Huijuan Zhang^4,5†^, Haixiao Jin^4,5^, Jin Guo^4,5^, Pan Liu^1,2^, Jiao Yang^1,2^, Zijian Wang^1,2^, Enmin Zhang^4,5^, Binbin Yu^1,2^, Liyuan Shi^1,2^, Jinrong He^4,5^, Peng Wang^1,2^, Jianchun Wei^4,5^, Youhong Zhong^1,2*^, Wei Li^4,5*^

^1^Yunnan Institute for Endemic Disease Control and Prevention, Dali, China

^2^Yunnan Key Laboratory for Zoonosis Control and Prevention, Dali, China

^3^School of Public Health, Dali University, Dali, China

^4^National Institute for Communicable Disease Control and Prevention (ICDC), China CDC, Beijing, China.

^5^National Key Laboratory of Intelligent Tracking and Forecasting for Infectious Diseases, Beijing, China

†These authors contributed equally to this study.

*** Correspondence:**Wei Li (ORCID: 0000-0002-3257-1540)
[liwei@icdc.cn](mailto:liwei@icdc.cn)
Youhong Zhong (ORCID: 0009-0004-5872-4746)
zyhong520@126.com

# Supplementary Figures and Tables

## Supplementary Figures


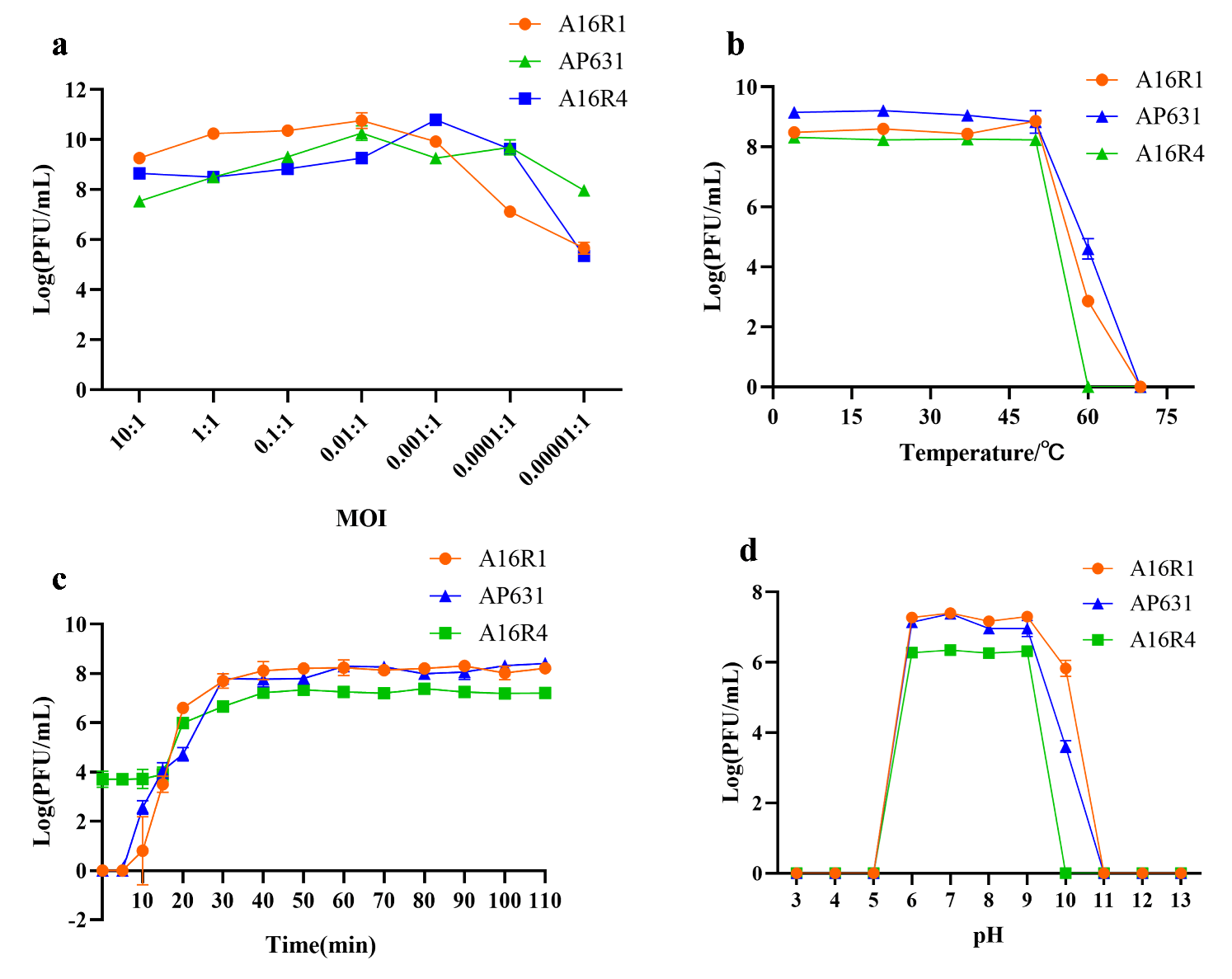


**Supplementary Figure 1** Optimal multiplicity of infection (MOI) of three phages (a); Thermal stability of three phages (b); One-step growth curves of three phages (c); Stability of three phages under pH3-13 (d).


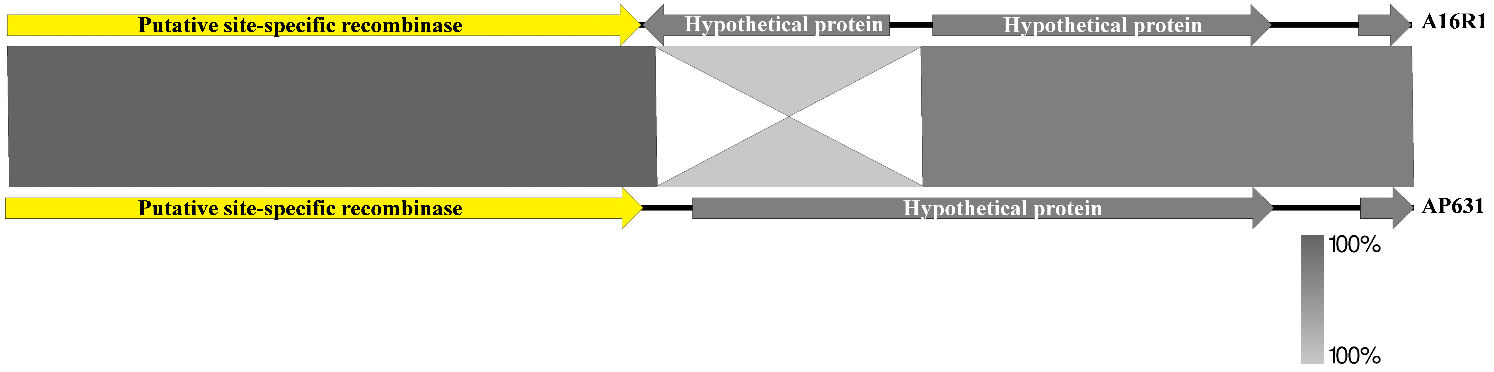


**Supplementary Figure 2**  Partial genome collinearity analysis of phages A16R1 and AP631.


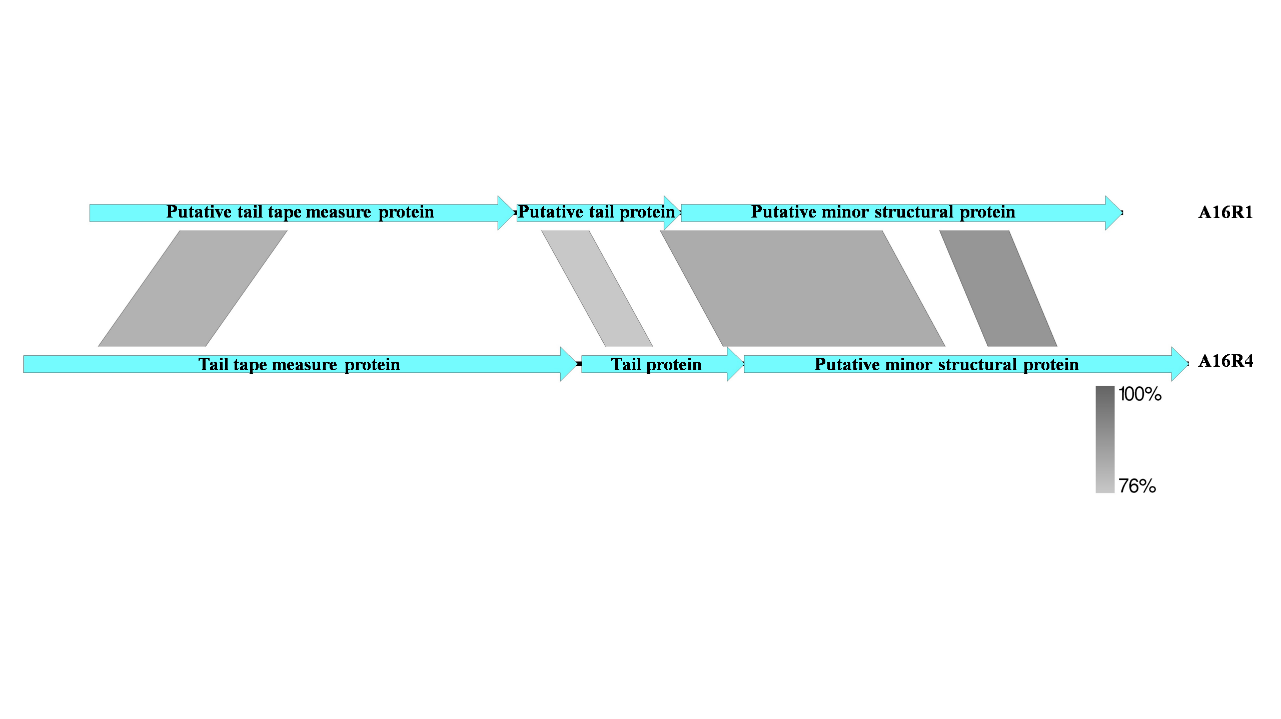


**Supplementary Figure 3** Tail structures collinearity analysis of A16R1 and A16R4.

## Supplementary Tables

**Supplementary Table 1** Protein function comparison results of A16R1.

| Coding Sequences | Length (bp) | Predicted function | The closest homolog in GenBank (GenBank acc. no.) | Coverage (%) | Identity (%) |
| --- | --- | --- | --- | --- | --- |
|  |  |  |  |  |  |
| ORF1 | 486 | Phage terminase, small subunit | Bacillus phage AP631 | 100 | 100 |
|  |  |  | YP_010739443.1 |  |  |
| ORF2 | 1698 | Phage terminase, large subunit | Bacillus phage AP631 | 100 | 100 |
|  |  |  | YP_010739444.1 |  |  |
| ORF3 | 1299 | Putative portal protein | Bacillus phage AP631 | 100 | 100 |
|  |  |  | YP_010739445.1 |  |  |
| ORF4 | 621 | Putative prohead protease | Bacillus phage AP631 | 100 | 100 |
|  |  |  | YP_010739446.1 |  |  |
| ORF5 | 1179 | Putative major capsid protein | Bacillus phage AP631 | 100 | 100 |
|  |  |  | YP_010739447.1 |  |  |
| ORF6 | 291 | Putative DNA packaging protein | Bacillus phage AP631 | 100 | 98.96 |
|  |  |  | YP_010739448.1 |  |  |
| ORF7 | 324 | Putative head-tail adaptor protein | Bacillus phage AP631 | 100 | 100 |
|  |  |  | YP_010739449.1 |  |  |
| ORF8 | 438 | Hypothetical protein | Bacillus phage AP631 | 100 | 100 |
|  |  |  | YP_010739450.1 |  |  |
| ORF9 | 360 | Structural protein | Bacillus phage AP631 | 100 | 100 |
|  |  |  | YP_010739451.1 |  |  |
| ORF10 | 609 | Putative major tail protein | Bacillus phage AP631 | 100 | 100 |
|  |  |  | YP_010739452.1 |  |  |
| ORF11 | 318 | Hypothetical protein | Bacillus phage AP631 | 100 | 100 |
|  |  |  | YP_010739453.1 |  |  |
| ORF12 | 177 | Hypothetical protein | Bacillus phage Gamma | 100 | 100 |
|  |  |  | YP_010739510.1 |  |  |
| ORF13 | 3852 | Putative tail tape measure protein | Bacillus phage AP631 | 100 | 100 |
|  |  |  | YP_010739455.1 |  |  |
| ORF14 | 1491 | Putative tail protein | Bacillus phage AP631 | 100 | 100 |
|  |  |  | YP_010739456.1 |  |  |
| ORF15 | 3999 | Putative minor structural protein | Bacillus phage AP631 | 100 | 99.92 |
|  |  |  | YP_010739457.1 |  |  |
| ORF16 | 426 | Holin | Bacillus phage AP631 | 100 | 100 |
|  |  |  | YP_010739458.1 |  |  |
| ORF17 | 702 | Lysin N-acetylmuramoyl-L-alanine amidase | Bacillus phage AP631 | 100 | 100 |
|  |  |  | YP_010739459.1 |  |  |
| ORF18 | 492 | Hypothetical protein | Bacillus phage AP631 | 100 | 100 |
|  |  |  | YP_010739460.1 |  |  |
| ORF19 | 213 | Hypothetical protein | Bacillus phage AP631 | 100 | 100 |
|  |  |  | YP_010739461.1 |  |  |
| ORF20 | 309 | Hypothetical protein | Bacillus phage AP631 | 100 | 100 |
|  |  |  | YP_010739462.1 |  |  |
| ORF21 | 183 | Hypothetical protein | Bacillus phage AP631 | 100 | 100 |
|  |  |  | YP_010739463.1 |  |  |
| ORF22 | 1182 | FtsK/SpoIIIE family protein | Bacillus phage Gamma | 100 | 100 |
|  |  |  | YP_010739520.1 |  |  |
| ORF23 | 528 | Hypothetical protein | Bacillus phage AP631 | 100 | 100 |
|  |  |  | YP_010739466.1 |  |  |
| ORF24 | 330 | Helix-turn-helix domain-containing protein | Bacillus phage vB_BanS_Booya | 100 | 95.41 |
|  |  |  | YP_010739799.1 |  |  |
| ORF25 | 120 | Hypothetical protein | Bacillus phage AP631 | 100 | 97.44 |
|  |  |  | YP_010739468.1 |  |  |
| ORF26 | 864 | Hypothetical protein | Bacillus phage AP631 | 100 | 100 |
|  |  |  | YP_010739469.1 |  |  |
| ORF27 | 1446 | Putative site-specific recombinase | Bacillus phage AP631 | 100 | 99.79 |
|  |  |  | YP_010739470.1 |  |  |
| ORF28 | 564 | Hypothetical protein | Bacillus phage AP631 | 94 | 98.87 |
|  |  |  | YP_010739471.1 |  |  |
| ORF29 | 774 | Hypothetical protein | Bacillus phage AP631 | 100 | 100 |
|  |  |  | YP_010739471.1 |  |  |
| ORF30 | 123 | Hypothetical protein | Bacillus phage AP631 | 100 | 100 |
|  |  |  | YP_010739472.1 |  |  |
| ORF31 | 348 | Helix-turn-helix Cro and cI family protein | Bacillus phage AP631 | 100 | 100 |
|  |  |  | YP_010739473.1 |  |  |
| ORF32 | 228 | Helix-turn-helix Cro and cI family protein | Bacillus phage AP631 | 100 | 100 |
|  |  |  | YP_010739474.1 |  |  |
| ORF33 | 183 | Hypothetical protein | Bacillus phage Wbeta | 98 | 96.61 |
|  |  |  | YP_459995.1 |  |  |
| ORF34 | 156 | Hypothetical protein | Bacillus phage AP631 | 100 | 100 |
|  |  |  | YP_010739476.1 |  |  |
| ORF35 | 828 | Putative antirepressor | Bacillus phage AP631 | 100 | 100 |
|  |  |  | YP_010739477.1 |  |  |
| ORF36 | 168 | Hypothetical protein | Bacillus phage AP631 | 100 | 100 |
|  |  |  | YP_010739478.1 |  |  |
| ORF37 | 441 | Hypothetical protein | Bacillus phage J5a | 100 | 97.95 |
|  |  |  | YP_010739933.1 |  |  |
| ORF38 | 939 | Putative replisome organizer protein | Bacillus phage AP631 | 97 | 100 |
|  |  |  | YP_010739480.1 |  |  |
| ORF39 | 912 | Putative DnaC protein | Bacillus phage AP631 | 100 | 100 |
|  |  |  | YP_010739481.1 |  |  |
| ORF40 | 234 | Hypothetical protein | Bacillus phage AP631 | 100 | 100 |
|  |  |  | YP_010739482.1 |  |  |
| ORF41 | 747 | RNA polymerase sporulation specific sigma factor SigF | Bacillus phage AP631 | 100 | 100 |
|  |  |  | YP_010739483.1 |  |  |
| ORF42 | 474 | Hypothetical protein | Bacillus phage AP631 | 100 | 100 |
|  |  |  | YP_010739484.1 |  |  |
| ORF43 | 543 | Hypothetical protein | Bacillus phage AP631 | 100 | 100 |
|  |  |  | YP_010739485.1 |  |  |
| ORF44 | 267 | Hypothetical protein | Bacillus phage AP631 | 100 | 100 |
|  |  |  | YP_010739486.1 |  |  |
| ORF45 | 144 | Hypothetical protein | Bacillus phage AP631 | 100 | 100 |
|  |  |  | YP_010739487.1 |  |  |
| ORF46 | 222 | Hypothetical protein | Bacillus phage AP631 | 100 | 100 |
|  |  |  | YP_010739488.1 |  |  |
| ORF47 | 189 | Hypothetical protein | Bacillus phage AP631 | 100 | 100 |
|  |  |  | YP_010739489.1 |  |  |
| ORF48 | 123 | Hypothetical protein | Bacillus phage AP631 | 100 | 100 |
|  |  |  | YP_010739490.1 |  |  |
| ORF49 | 408 | Transcriptional regulator | Bacillus phage Wbeta | 100 | 98.52 |
|  |  |  | YP_460009.1 |  |  |
| ORF50 | 522 | Hypothetical protein | Bacillus phage AP631 | 100 | 100 |
|  |  |  | YP_010739492.1 |  |  |
| ORF51 | 267 | Hypothetical protein | Bacillus phage AP631 | 100 | 100 |
|  |  |  | YP_010739493.1 |  |  |
| ORF52 | 222 | Hypothetical protein | Bacillus phage AP631 | 100 | 100 |
|  |  |  | YP_010739494.1 |  |  |
| ORF53 | 255 | Transcription factor tflle subunit | Bacillus phage vB_BanS_McSteamy | 97 | 93.9 |
|  |  |  | YP_010739771.1 |  |  |
| ORF54 | 171 | Hypothetical protein | Bacillus phage AP631 | 100 | 100 |
|  |  |  | YP_010739496.1 |  |  |
| ORF55 | 414 | Hypothetical protein | Bacillus phage AP631 | 100 | 100 |
|  |  |  | YP_010739497.1 |  |  |
| ORF56 | 384 | Putative endonuclease | Bacillus phage AP631 | 100 | 100 |
|  |  |  | YP_010739498.1 |  |  |

**Supplementary Table 2** Protein function comparison results of A16R4.

| Coding Sequences | Length (bp) | Predicted function | The closest homolog in GenBank (GenBank acc. no.) | Coverage (%) | Identity (%) |
| --- | --- | --- | --- | --- | --- |
|  |  |  |  |  |  |
| ORF1 | 159 | Hypothetical protein | Bacillus phage phiS58 | 98 | 51.92 |
|  |  |  | ALO79941.1 |  |  |
| ORF2 | 168 | Hypothetical protein | Bacillus phage vB_BtS_BMBtp16 | 96 | 62.26 |
|  |  |  | ALF01622.1 |  |  |
| ORF3 | 252 | Transposase | Bacillus phage PfEFR-5 | 93 | 51.28 |
|  |  |  | YP_009285287.1 |  |  |
| ORF4 | 483 | Nucleotide pyrophosphohydrolase | Bacillus phage BVE2 | 100 | 94.38 |
|  |  |  | AUG88584.1 |  |  |
| ORF5 | 270 | Hypothetical protein | Bacillus phage 11143 | 100 | 100 |
|  |  |  | ADA84980.1 |  |  |
| ORF6 | 651 | Hypothetical protein | Bacillus phage SBSphiJ7 | 81 | 51.85 |
|  |  |  | UPI13461.1 |  |  |
| ORF7 | 675 | YopX protein | Bacillus phage 11143 | 100 | 100 |
|  |  |  | ADA84957.1 |  |  |
| ORF8 | 168 | Hypothetical protein | Bacillus phage PfEFR-4 | 100 | 100 |
|  |  |  | YP_009830783.1 |  |  |
| ORF9 | 264 | Hypothetical protein | Bacillus phage vB_BceS-MY192 | 100 | 100 |
|  |  |  | YP_009830066.1 |  |  |
| ORF10 | 249 | Transcriptional regulator | Bacillus phage vB_BceS-MY192 | 100 | 100 |
|  |  |  | YP_009830067.1 |  |  |
| ORF11 | 99 | Hypothetical protein | Bacillus phage vB_BceS-MY192 | 100 | 100 |
|  |  |  | YP_009830068.1 |  |  |
| ORF12 | 132 | DUF3983 domain-containing protein | Bacillus phage PfEFR-5 | 100 | 97.67 |
|  |  |  | YP_009285303.1 |  |  |
| ORF13 | 171 | Hypothetical protein | Bacillus phage PfEFR-5 | 100 | 99.7 |
|  |  |  | YP_009285304.1 |  |  |
| ORF14 | 474 | Transcriptional activator | Bacillus phage PfEFR-5 | 100 | 100 |
|  |  |  | YP_009285305.1 |  |  |
| ORF15 | 543 | Integrase | Bacillus phage PfEFR-5 | 100 | 100 |
|  |  |  | YP_009285306.1 |  |  |
| ORF16 | 243 | Hypothetical protein | Bacillus phage PfEFR-5 | 47 | 89.47 |
|  |  |  | YP_009285307.1 |  |  |
| ORF17 | 375 | Hypothetical protein | Bacillus phage vB_BtS_BMBtp3 | 83 | 85.44 |
|  |  |  | YP_009193976.1 |  |  |
| ORF18 | 255 | Hypothetical protein | Stenotrophomonas phage TS-12 | 100 | 98.81 |
|  |  |  | QTJ63065.1 |  |  |
| ORF19 | 213 | Hypothetical protein | Stenotrophomonas phage TS-12 | 100 | 100 |
|  |  |  | QTJ63064.1 |  |  |
| ORF20 | 174 | Hypothetical protein | Stenotrophomonas phage TS-12 | 100 | 100 |
|  |  |  | QTJ63063.1 |  |  |
| ORF21 | 363 | HNH endonuclease | Stenotrophomonas phage TS-12 | 100 | 100 |
|  |  |  | QTJ63062.1 |  |  |
| ORF22 | 381 | Terminase small subunit | Bacillus phage PfEFR-5 | 100 | 100 |
|  |  |  | YP_009285249.1 |  |  |
| ORF23 | 1776 | Terminase large subunit | Bacillus phage PfEFR-5 | 100 | 99.49 |
|  |  |  | YP_009285250.1 |  |  |
| ORF24 | 1215 | Portal protein | Bacillus phage vB_BceS-MY192 | 100 | 100 |
|  |  |  | YP_009830079.1 |  |  |
| ORF25 | 756 | Head maturation protease | Bacillus phage vB_BceS-MY192 | 100 | 99.6 |
|  |  |  | YP_009830080.1 |  |  |
| ORF26 | 1188 | Major capsid protein | Bacillus phage PfEFR-5 | 100 | 100 |
|  |  |  | YP_009285253.1 |  |  |
| ORF27 | 276 | Head-tail adaptor Ad1 | Bacillus phage PfEFR-5 | 100 | 98.9 |
|  |  |  | YP_009285254.1 |  |  |
| ORF28 | 348 | Head closure Hc1 | Bacillus phage PfEFR-5 | 100 | 99.13 |
|  |  |  | YP_009285255.1 |  |  |
| ORF29 | 438 | HK97 gp10 family protein | Bacillus phage PfEFR-5 | 100 | 100 |
|  |  |  | YP_009285256.1 |  |  |
| ORF30 | 360 | Putative structural protein | Bacillus phage PfEFR-5 | 100 | 99.16 |
|  |  |  | YP_009285257.1 |  |  |
| ORF31 | 585 | Major tail protein | Bacillus phage PfEFR-5 | 100 | 100 |
|  |  |  | YP_009285258.1 |  |  |
| ORF32 | 396 | Head-tail adaptor | Bacillus phage vB_BceS-MY192 | 100 | 100 |
|  |  |  | YP_009830087.1 |  |  |
| ORF33 | 141 | Hypothetical protein | Bacillus phage PfEFR-5 | 100 | 100 |
|  |  |  | YP_009285260.1 |  |  |
| ORF34 | 5016 | Tail tape measure protein | Bacillus phage PfEFR-5 | 100 | 99.64 |
|  |  |  | YP_009285261.1 |  |  |
| ORF35 | 1476 | Tail protein | Bacillus phage phIS3501 | 99.99 | 78.73 |
|  |  |  | YP_007004373.1 |  |  |
| ORF36 | 4026 | Putative minor structural protein | uncultured Caudovirales phage | 99 | 92.96 |
|  |  |  | ASN69602.1 |  |  |
| ORF37 | 240 | Putative peptidase | Bacillus phage vB_BanS_Athena | 100 | 100 |
|  |  |  | YP_010742666.1 |  |  |
| ORF38 | 144 | Putative holin | Bacillus phage BtCS33 | 100 | 93.62 |
|  |  |  | YP_006488687.1 |  |  |
| ORF39 | 798 | Putative N-acetylmuramoyl-L-alanine amidase | Bacillus phage vB_BanS_Athena | 100 | 100 |
|  |  |  | YP_010742668.1 |  |  |
| ORF40 | 207 | Helix-turn-helix domain-containing protein | Bacillus phage PfEFR-5 | 100 | 100 |
|  |  |  | YP_009285269.1 |  |  |
| ORF41 | 393 | Hypothetical protein | Bacillus phage PfEFR-5 | 100 | 100 |
|  |  |  | YP_009285270.1 |  |  |
| ORF42 | 327 | Hypothetical protein | Bacillus phage PfEFR-5 | 100 | 100 |
|  |  |  | YP_009285271.1 |  |  |
| ORF43 | 1149 | FtsK/SpoIIIE-like protein | Bacillus phage vB_BceS-MY192 | 100 | 100 |
|  |  |  | YP_009830100.1 |  |  |
| ORF44 | 636 | Replication initiation protein | Bacillus phage vB_BceS-MY192 | 100 | 99.53 |
|  |  |  | YP_009830101.1 |  |  |
| ORF45 | 216 | Hypothetical protein | Bacillus phage vB_BceS-MY192 | 100 | 100 |
|  |  |  | YP_009830102.1 |  |  |
| ORF46 | 594 | Hypothetical protein | Stenotrophomonas phage TS-12 | 100 | 100 |
|  |  |  | QTJ63035.1 |  |  |
| ORF47 | 1110 | Putative integrase | Bacillus phage vB_BceS-MY192 | 86 | 100 |
|  |  |  | YP_009830040.1 |  |  |
| ORF48 | 1194 | Transcriptional regulator | Bacillus phage vB_BceS-MY192 | 100 | 100 |
|  |  |  | YP_009830041.1 |  |  |
| ORF49 | 135 | Hypothetical protein | Bacillus phage vB_BceS-MY192 | 100 | 100 |
|  |  |  | YP_009830042.1 |  |  |
| ORF50 | 354 | Transcriptional repressor | Bacillus phage vB_BceS-MY192 | 100 | 99.15 |
|  |  |  | YP_009830043.1 |  |  |
| ORF51 | 186 | Transcriptional regulator | Bacillus phage vB_BceS-MY192 | 100 | 100 |
|  |  |  | YP_009830044.1 |  |  |
| ORF52 | 297 | Helix-turn-helix domain-containing protein | Bacillus phage vB_BceS-MY192 | 100 | 100 |
|  |  |  | YP_009830045.1 |  |  |
| ORF53 | 165 | Hypothetical protein | Bacillus phage vB_BceS-MY192 | 100 | 100 |
|  |  |  | YP_009830046.1 |  |  |
| ORF54 | 177 | Hypothetical protein | Bacillus phage vB_BceS-MY192 | 100 | 100 |
|  |  |  | YP_009830047.1 |  |  |
| ORF55 | 879 | DnaD-like helicase loader | Bacillus phage vB_BceS-MY192 | 100 | 99.32 |
|  |  |  | YP_009830048.1 |  |  |
| ORF56 | 915 | DnaC-like helicase loader | Bacillus phage vB_BceS-MY192 | 100 | 100 |
|  |  |  | YP_009830049.1 |  |  |
| ORF57 | 195 | Hypothetical protein | Bacillus phage BVE2 | 100 | 90.62 |
|  |  |  | AUG88588.1 |  |  |
| ORF58 | 279 | Transcription state regulatory protein abrB | Bacillus phage phBC6A51 | 96 | 51.65 |
|  |  |  | NP_852518.1 |  |  |
| ORF59 | 120 | Hypothetical protein | - | - | - |
|  |  |  | - |  |  |

**Supplementary Table 3** Bacteria of host spectrum information.

| **Strains** | | **Origin (ID)** | **Identification** | **Strains** | | **Origin (ID)** | **Identification** |
| --- | --- | --- | --- | --- | --- | --- | --- |
| *Bacillus anthracis* (6) | Kunming | Preserved in our laboratory | Separate identification according to national standards WS 283-2008 (China) | *Bacillus thuringiensis* (16) | BT01 | Preserved in our laboratory | Separate identification according to national standards GB 4789. 14—2014 (China) |
|  | Chongming | Preserved in our laboratory | Separate identification according to national standards WS 283-2008 (China) |  | BT02 | Preserved in our laboratory | Separate identification according to national standards GB 4789. 14—2014 (China) |
|  | Dongchuan | Preserved in our laboratory | Separate identification according to national standards WS 283-2008 (China) |  | BT03 | Preserved in our laboratory | Separate identification according to national standards GB 4789. 14—2014 (China) |
|  | Midu | Preserved in our laboratory | Separate identification according to national standards WS 283-2008 (China) |  | BT04 | Preserved in our laboratory | Separate identification according to national standards GB 4789. 14—2014 (China) |
|  | Huize | Preserved in our laboratory | Separate identification according to national standards WS 283-2008 (China) |  | BT05 | Preserved in our laboratory | Separate identification according to national standards GB 4789. 14—2014 (China) |
|  | A16R | Preserved in our laboratory | [1] |  | BT06 | From ACCC  (ACCC 10073) | - |
| *Bacillus cereus* (28) | CMCC 63301 | From CMCC | - |  | BT07 | From ACCC  (ACCC 10018) | - |
|  | ATCC 13061 | From ATCC | - |  | BT08 | From ACCC  (ACCC 10022) | - |
|  | ATCC 10876 | From ATCC | - |  | BT09 | From ACCC  (ACCC 10321) | - |
|  | ATCC 14579 | From ATCC | - |  | BT10 | From ACCC  (ACCC 10308) | - |
|  | ATCC 11778 | From ATCC | - |  | BT11 | From ACCC  (ACCC 10314) | - |
|  | BC04 | Soil | Separate identification according to national standards GB 4789. 14—2014 (China) |  | BT12 | From ACCC  (ACCC 10301) | - |
|  | BC07 | Soil | Separate identification according to national standards GB 4789. 14—2014 (China) |  | BT13 | From CGMCC  (CGMCC 1.15822) | - |
|  | BC31 | Soil | Separate identification according to national standards GB 4789. 14—2014 (China) |  | BT14 | From ATCC  (ATCC 39756) | - |
|  | BC45 | Soil | Separate identification according to national standards GB 4789. 14—2014 (China) |  | BT15 | From DSM  (DSM 6070) | - |
|  | BC46 | Soil | Separate identification according to national standards GB 4789. 14—2014 (China) |  | BT16 | From DSM  (DSM 2046) | - |
|  | BC98 | Food | Separate identification according to national standards GB 4789. 14—2014 (China) | *Bacillus subtilis* (8) | BS02 | Preserved in our laboratory | Separate and identify according to relevant national standards |
|  | BC104 | Food | Separate identification according to national standards GB 4789. 14—2014 (China) |  | BS03 | Preserved in our laboratory | Separate and identify according to relevant national standards |
|  | BC105 | Food | Separate identification according to national standards GB 4789. 14—2014 (China) |  | ATCC 6633 | From ATCC | - |
|  | BC108 | Food | Separate identification according to national standards GB 4789. 14—2014 (China) |  | ATCC 9372 | From ATCC | - |
|  | BC111 | Food | Separate identification according to national standards GB 4789. 14—2014 (China) |  | A383 | Beijing Hospital | - |
|  | BC112 | Food | Separate identification according to national standards GB 4789. 14—2014 (China) |  | A174 | Beijing Hospital | - |
|  | BC113 | Food | Separate identification according to national standards GB 4789. 14—2014 (China) |  | A175 | Beijing Hospital | - |
|  | BC248 | Food | Separate identification according to national standards GB 4789. 14—2014 (China) |  | A850 | Beijing Hospital | - |
|  | BC273 | Food | Separate identification according to national standards GB 4789. 14—2014 (China) | *Bacillus mycoides* (4) | BMY01 | From CGMCC  (CGMCC 1.865) | - |
|  | BC293 | Food | Separate identification according to national standards GB 4789. 14—2014 (China) |  | BMY02 | From ACCC  (ACCC 10237) | - |
|  | BC334 | Animal intestines | Separate identification according to national standards GB 4789. 14—2014 (China) |  | BMY03 | From ACCC  (ACCC 10264) | - |
|  | BC335 | Soil | Separate identification according to national standards GB 4789. 14—2014 (China) |  | BMY04 | From CGMCC  (CMGCC 1.10168) | - |
|  | BC336 | Soil | Separate identification according to national standards GB 4789. 14—2014 (China) | *Bacillus licheniformis* (3) | ATCC 12759 | From ATCC | - |
|  | BC337 | Soil | Separate identification according to national standards GB 4789. 14—2014 (China) |  | BL01 | Preserved in our laboratory | Separate and identify according to relevant national standards |
|  | BC338 | Soil | Separate identification according to national standards GB 4789. 14—2014 (China) |  | BL02 | Preserved in our laboratory | Separate and identify according to relevant national standards |
|  | BC553 | Food | Separate identification according to national standards GB 4789. 14—2014 (China) | *Bacillus pumilus* (2) | A784 | Beijing Hospital | - |
|  | BC558 | Food | Separate identification according to national standards GB 4789. 14—2014 (China) |  | BP02 | Preserved in our laboratory | Separate and identify according to relevant national standards |
|  | BC574 | Food | Separate identification according to national standards GB 4789. 14—2014 (China) | *Bacillus megaterium* (2) | BMG01 | From CGMCC (CGMCC1.217) | - |
| *Bacillus aerius* (1) | A480 | Beijing Hospital | - |  | BMG02 | Preserved in our laboratory | Separate and identify according to relevant national standards |
| *Bacillus sphaericus* (1) | A184 | Beijing Hospital | - | *Bacillus* (8) | A1280 | Beijing Hospital | - |
| *Oceanobacillus sojae* (1) | A616 | Beijing Hospital | - |  | A1266 | Beijing Hospital | - |
| *Geobacillus stearothermophilus* (1) | A197 | Beijing Hospital | - |  | A1282 | Beijing Hospital | - |
| *Bacillus cytotoxicus* (1) | Bcyt01 | From ACCC  (ACCC 10402) | - |  | A859 | Beijing Hospital | - |
| *Bacillus pseudomycoides* (1) | Bpseu01 | From ACCC  (ACCC 10238) | - |  | A1281 | Beijing Hospital | - |
| *Bacillus wiedmannii* (1) | Bwied01 | From DSM  (DSM 102050) | - |  | A287 | Beijing Hospital | - |
| *Bacillus gaemokensis* (1) | Bgae01 | From JCM  (JCM 15801) | - |  | A648 | Beijing Hospital | - |
| *Bacillus manliponensis* (1) | Bman01 | From DSM  (DSM 26473) | - |  | A170 | Beijing Hospital | - |
| *Bacillus bingmayongensis* (1) | Bbmy01 | From CGMCC  (CGMCC 1.12043) | - | *Bacillus weihenstephanensis* (2) | Bwei01 | From ACCC  (ACCC 01508) | - |
| *Bacillus toyonensis* (1) | Btoyo01 | From NCIMB  (NCIMB 14858) | - |  | Bwei02 | From ACCC  (ACCC 01965) | - |

（Notes: American type culture collection, ATCC (<https://www.atcc.org/>). Agricultural Culture Collection of China, ACCC (<https://www.iarrp.cn/en/aboutus/structure/scientific/287900.htm>). China General Microbiological Culture Collection Center, CGMCC (<https://cgmcc.net/>). German Collection of Microorganisms and Cell Cultures, DSMZ (<https://www.dsmz.de/>). National Collections of Industrial, Food and Marine Bacterial, NCIMB (<https://www.ncimb.com/culture-collection/>). Japan collection of microorganisms, JCM (<https://jcm.brc.riken.jp/en/>).

1. Xiankai Liu XQ,  Li Zhu, Dongshu Wang, Zhiqi Gao, Haijun Deng, Weili Wu, Tao Hu, Chen Chen, Weijun Chen, Hengliang Wang (2015) Genome sequence of Bacillus anthracis attenuated vaccine strain A16R used for human in China. J Biotechnol 210:15-16
